# Astrocyte Infection Is Required for Retrovirus-Induced Spongiform Neurodegeneration Despite Suppressed Viral Protein Expression

**DOI:** 10.3389/fnins.2019.01166

**Published:** 2019-10-29

**Authors:** Sandra M. Cardona, Jaclyn M. Dunphy, Alvin S. Das, Connor R. Lynch, William P. Lynch

**Affiliations:** ^1^Department of Integrative Medical Sciences, Northeast Ohio Medical University, Rootstown, OH, United States; ^2^Program in Cellular and Molecular Biology, School of Biomedical Sciences, Kent State University, Kent, OH, United States; ^3^Program in Neuroscience, School of Biomedical Sciences, Kent State University, Kent, OH, United States; ^4^Brain Health Research Institute, Kent State University, Kent, OH, United States

**Keywords:** retrovirus, neurotropism, glia, astrocytes, neural stem cells, neural progenitor cells, spongiform neurodegeneration, virus suppression

## Abstract

The ability of retroviruses (RVs) to cause neurodegeneration is critically dependent upon two activities of the envelope protein (Env). First, Env facilitates viral genome delivery to CNS target cells through receptor binding and membrane fusion. Second, Env expression within one or more targets indirectly alters the physiology of certain neurons. Although the major Env expressing CNS cell types have been identified for many neurovirulent RVs, it remains unresolved, which targets play a causal role in neuropathogenesis. Moreover, this issue is complicated by the potential for post-infection virus suppression. To address these questions we explored herein, whether and how cryptic neurotropism differences between ecotropic and amphotropic murine leukemia viruses (MLVs) impacted neurovirulence. Neurotropism was first explored *ex vivo* using (1) acute primary glial cell cultures and (2) neural progenitor cell (NPC)- neural stem cell (NSC) neural sphere (NPH) chimeras. These experiments indicated that primary astrocytes and NPCs acutely restrict amphotropic but not ecotropic virus entry. CNS tropism was investigated using NSC transplant-based Cre-vector pseudotyping wherein mTmG transgenic fluorescent protein reporter mice revealed both productive and suppressed infection. Cre-pseudotyping with FrCasE, a prototypic neurovirulent ecotropic virus, identified glia and endothelia, but not neurons, as targets. Almost two-thirds (62%) of mGFP+ cells failed to show Env expression, suggesting widespread virus suppression. To circumvent RV superinfection interference confounds, targets were also identified using ecotropic packaging NSCs. These experiments identified known ecotropic targets: microglia, oligodendrocyte progenitor cells (OPCs) and endothelia. Additionally, one third of mGFP+ cells were identified as protoplasmic astrocytes, cells that rarely express virus *in vivo*. A CNS targeting comparison between isogenic ecotropic (FrCasE) and amphotropic (FrAmE) viruses showed a fourfold higher astrocyte targeting by FrCasE. Since ecotropic Env pseudotyping of amphotropic virus in the CNS dramatically exacerbates neurodegeneration, these results strongly suggest that astrocyte infection is a major disease requirement. Moreover, since viral Env protein expression is largely subdetectable in astrocytes, minimal viral protein expression appears sufficient for affecting neuronal physiology. More broadly, these findings raise the specter that subdetectable astrocyte expression of exogenous or endogenous RVs could play a major role in human and animal neurodegenerative diseases.

## Introduction

A variety of exogenous human and animal retroviruses (RVs) are capable of causing progressive neurodegenerative diseases characterized by a loss of cognitive and/or motor function. In addition, human endogenous RVs have been implicated in the pathogenesis of sporadic amyotrophic lateral sclerosis (Li et al., 2015), multiple sclerosis (Perron et al., 1997, 2012a), and schizophrenia (Perron et al., 2012b). In order to understand how RVs cause neurodegeneration, murine leukemia viruses (MLVs) have historically served as tractable model systems since their discovery as neurodegenerative agents in the 1970s. Moreover, specific viral clones have been characterized that are capable of causing rapid, progressive, paralytic diseases accompanied by non-inflammatory spongiform neuropathology reminiscent of prion diseases (Gardner et al., 1973; Gardner, 1978, 1985). Prior research has identified many different CNS cell types as being infected by both neurovirulent and non-neurovirulent MLVs (Baszler and Zachary, 1991; Lynch et al., 1991; Gravel et al., 1993; Lynch and Portis, 1993; Robertson et al., 1997), but it is unknown which, if any of these cells is responsible for the altered neurophysiology and clinical neurological changes arising in infected mice (Li et al., 2014). While postnatally proliferating neurons are MLV targets, they do not undergo neurodegenerative changes (Lynch et al., 1991; Gravel et al., 1993). Moreover, neurovirulent MLVs do not appear to infect the degenerating motor system neurons (Kay et al., 1991; Lynch et al., 1991; Gravel et al., 1993; Li et al., 2014). Importantly, MLVs require cell division in order for the reverse transcribed proviral DNA to integrate in the host genome and be expressed (Kay et al., 1991; Lynch et al., 1991). Because motor neuron cell division ceases during midgestation, well prior to retrovirus exposure during the neonatal period, their infection is precluded. Instead, MLV infection of postnatally proliferating vascular cells and glia appears to mediate disease (Gardner et al., 1973; Brooks et al., 1979; Pitts et al., 1987; Baszler and Zachary, 1990, 1991; Morey and Wiley, 1990; Lynch et al., 1991; Hoffman et al., 1992; Nagra et al., 1992; Masuda et al., 1993), although the mechanisms remain unknown. Genetic mapping studies have identified the viral *env* gene as encoding the major neurovirulence determinants (DesGroseillers et al., 1984; Portis et al., 1990, 1995; Wong and Yuen, 1992), and neural stem cell (NSC)-based brain chimera studies have demonstrated that the virus need only encode the Env gene to induce neuropathogenic changes (Li et al., 2011). However, experiments aimed at understanding the effect of neurovirulent Env expression on specific glial cell subtypes has been challenging owing to the difficulty in generating Env transgenic mice that develop acute disease. As an alternative strategy, our laboratory has used stem cell-based brain chimeras to assess how viral protein expression affects the CNS. These experiments showed that high level CNS expression of neurovirulent Env from engrafted C17.2 NSCs was not sufficient to cause spongiosis (Lynch et al., 1996). Instead, spongiform neurodegeneration was only observed when engrafted NSCs delivered Env-encoding virus to endogenous host cells, however, the identification of the cellular targets critical for disease development could not be discerned.

Important preliminary insight into the nature of the critical CNS targets was gained from investigations exploring the neurovirulence potential of various MLV tropism groups. Historically, viral tropism refers to a classification of RVs based on the species that they infect, which was later defined at the molecular level based on the specific cell surface proteins used by the RV Env for entry. In this regard, ecotropic viruses infect mice and rats, and their Env proteins bind and enter cells via the murine cationic amino acid transporter-1 (mCAT-1). CasBrE is an example of a neurovirulent ecotropic RV, whereas the Friend virus is a non-neurovirulent ecotropic virus. In contrast, amphotropic RVs infect a variety of mammalian hosts including mice and humans, with Env binding and entry via the sodium dependent phosphate transporter-2 (PiT2). Amphotropic viruses (such as clone 4070A) were widely reported to not cause spongiform neurodegeneration nor clinical neurological disease in commonly used laboratory mouse strains (Rasheed et al., 1976; DesGroseillers et al., 1984; Gardner, 1991; Jolicoeur et al., 1992). Moreover, attempts to exacerbate or amplify any neurovirulence by placing its *env* gene into neurovirulent or neuroinvasive virus backgrounds, or by NSC-directed delivery to the CNS failed to reveal any significant neuropathogenic potential (Traister and Lynch, 2002). However, Munk et al. (1997) observed spongiform neuropathology and neurological disease in some less commonly used mouse strains after neonatal infection with a chimeric amphotropic virus. In this virus, named MoAmphoV, the 4070A *env* gene replaced the ecotropic *env* gene of Moloney MLV (Munk et al., 1997). Importantly, the MoAmphoV-induced neurological disease was exacerbated when mice were co-infected with Friend MLV. These findings suggested that ecotropic viral pseudotyping was expanding amphotropic neurotropism. Direct proof that ecotropic Env pseudotyping of amphotropic virus facilitated acute spongiform neurodegeneration in otherwise resistant mice was carried out by transplantation of 4070A-infected NSCs co-expressing either CasBrE or Friend ecotropic Envs from non-packaged vectors (Li et al., 2011). Interestingly, 4070A CNS cellular tropism differences could not be detected with ecotropic Env pseudotyping, despite dramatic differences in neuropathology. Because the identification of infected CNS cell types in that analysis was dependent upon the detection of viral gene products with specific antibody probes, any cell type that suppressed virus expression would have been excluded. In this regard, we have recently reported that *in vivo*, engrafted primary neural progenitor cells (NPCs) suppress virus/Env expression upon differentiation into astrocytes (Li et al., 2016). This finding is consistent with reports of a very low frequency of astrocyte infection/virus expression noted in MLV- and human RV-induced diseases (cf. Lynch et al., 1991; Gravel et al., 1993; Gorry et al., 1999). The idea that subdetectable virus expression could play a major role in viral neuropathogenesis seems at first incongruous; however, given the capacity of certain retroviruses like CasBrE to dramatically increase neuronal excitability (Li et al., 2014), it may be critically important for astroglial cells to control the expression of exogenous and endogenous RV protein expression.

Herein, we examine the idea that amphotropic virus infection of CNS cell types critical for neurodegeneration is restricted. Moreover, we explore whether when neurovirulent virus infection of the restricted cell type does occur, it is accompanied Env protein/virus suppression. To address these questions we employed novel *in vitro* and *in vivo* strategies to identify “cryptic” CNS viral targets, and establish that these cryptic targets account for the neurotropism and neurovirulence differences observed between ecotropic and amphotropic Envs. These investigations strongly implicate astrocytes as the obscure critical cryptic target. Given the emerging understanding of the role that astrocytes play in regulating synaptic communications (Perea et al., 2009), these findings raise interesting new questions about the mechanisms by which viral Env proteins may influence critical neuron–glial interactions.

## Materials and Methods

### Virus

Ecotropic FrCasE, Fr57E and amphotropic 4070A, FrAmE viruses were generated by transfection of proviral plasmid into Mus dunni or NIH3T3 cells and the resulting tissue culture supernatants were titered by virus titration assay (VTA) (Czub et al., 1991; Traister and Lynch, 2002). Cre-recombinase (Cre) encoding pseudotyped virus was generated by transfection of PT67 cells with the Cre and puromycin-*N*-acetyl transferase (puro) encoding retroviral vector, FINT#22 (Figure 3A; Westerman and Leboulch, 1996), followed by selection with puromycin and collection of the supernatant after growth of cells in the absence of selection.

### Virus Titration Assay (VTA)

Virus titers on tissue culture supernatants from C17.2 NSCs infected with FrCasE, Fr57E, 4070A or FrAmE were performed previously as outlined (Czub et al., 1991; Traister and Lynch, 2002). Cre encoding virus titers were determined using VTAs wherein *mus dunni* target cells exposed to viral stocks were selected with puromycin at 4 μg/ml of medium followed by colony counting 1 week later (Westerman and Leboulch, 1996).

### Cells

C17.2 NSCs were grown in Dulbecco’s modified eagle medium (DMEM) supplemented with 10% fetal bovine serum (DM10F) as previously described (Lynch et al., 1996). C17.2 NSCs expressing the Cre-recombinase retroviral vector, FINT#22, were generated by two rounds of infection with PT67 supernatants in the presence of 8 μg/ml of polybrene, and selected with puromycin for 1 week. Puromycin resistant C17.2 cells, named VFCs, were subsequently infected with FrCasE, Fr57E, 4070A or FrAmE viruses. Alternatively, Fint#22 was transfected into C17.2 NSCs, cells selected for puromycin resistance, and were then infected with FrCasE, Fr57E, 4070A, or FrAmE virus stocks. C17.2 cells expressing CasBrE Env from a non-packaging vector (lacking the psi sequence) were generated as previously outlined (Li et al., 2011) using the 15-1EIH plasmid followed by selection with hygromycin. Packaging/producer VFCs were generated from 15-1EIH NSCs that were transfected with p*gagpolgpt* as outlined previously (Li et al., 2011) and subsequently infected with Cre encoding virus from PT67 cells and selected for puromycin resistance.

Primary CNS glial cultures were generated from P0-1 IRW neonatal cortices dissociated into single cells using Miltenyi dissociation Kit (Papain). Briefly, cryoanesthesized mice were decapitated, brains were removed and cortices were dissected and placed on ice-cold Hank’s buffered salt solution without calcium chloride (HBSS w/o Ca^2+^). Cortices were transferred to a petri dish for the removal of meninges under a dissecting microscope. Meninges-free cortices were minced into small pieces using heat-sterilized razor blades and triturated by pipetting with fire polished pasteur pipets. Brain suspensions were transferred to tubes containing HBSS w/o Ca^2+^ and spun down for 10 min at 800 rpm, 4°C. Upon gentle removal of the supernant, pelleted brains pieces were resuspended in enzyme mix 1, and incubated for 10 min in a 37°C water bath. Mechanical trituration was done using fire polished glass pasteur pipettes, followed by addition of the papain enzyme mix 2 and 20-min incubation with agitation and mechanical trituration to improve the dissociation of brain tissue into single cells. Treated cells were rinsed twice with HBSS supplemented with calcium chloride (HBSS w/Ca^2+^).

Once dissociated the primary CNS cells were acutely exposed to ecotropic, amphotropic, eco-pseudotyped amphotropic virus or control cell supernatants at a multiplicity of infection (MOI) of 1, by spinoculation in 6-well primaria plates. Cells were grown for various periods as indicated in the results section in Advanced DMEM, 5% fetal bovine serum, 1% penicillin, streptomycin and 10 μg/mL EGF, followed by fixation in 3.7% formaldehyde in PBS for 5–10 min.

Neural progenitor cells from F1 mTmG × IRW strain mice were obtained from P0-1 brains as described previously (Li et al., 2016). Briefly, neonatal cortices were dissociated as indicated above for primary glial cultures, but after rinsing in HBSS w/Ca^2+^ they were placed in DMEM/F12 medium with N2 supplement and basic fibroblast growth factor (b-FGF), EGF, penicillin and streptomycin, and grown in suspension as NPHs in T25 flasks (Nunc). NPHs were dissociated into single cells every 3 days for 4–5 passages at which time they were dissociated, resuspended at approximately 5 × 10^6^ cells/ml in NPH medium with 10% dimethyl sulfoxide (DMSO) and frozen at −140°C for later use in NPC/NSC chimera assays. The NPCs stocks utilized were greater than 98% positive for nestin and NG2, consistent with our previous studies on cells that could engraft and differentiate after CNS transplantation (Li et al., 2016).

### NPC/NSC NPH Chimera Cre Virus Transduction Assay

To assay the capacity of VFCs to pseudotype and transduce the Cre encoding FINT#22 vector to readout mTmG NPCs, chimeric NPH between NSC and NPCs were established by mixing 10,000 C17.2 VFCs with ∼5 × 10^5^ mTmG NPCs in 1 ml of complete NPH medium (serum-free) in each well of TC12 plates (Thermo Scientific). VFCs used were from actively growing attached cultures, which had been previously characterized for MLV production. VFCs were trypsinized, dissociated into single cells by trituration and rinsed in DMEM/F12 without additives prior to mixing with NPCs. NPCs were freshly thawed from frozen −140°C stocks, rinsed with DMEM/F12 medium and then aliquotted into the TC12 plates. Within 24 h spheres of more than 10 cells could be recognized arising from cell–cell attachment and ongoing cell division. By 3 days in culture 20–50 NPHs between 10 and 100 μm in diameter could be observed in each microscope field (100x final mag). A NPH was scored as positive if it had one or more mGFP+ cells. Five or more randomly chosen microscope fields were sampled in each assayed well. The number of GFP+ NPHs was divided by the total number of mTomato+ NPHs in the microscope fields surveyed. Assays were performed in triplicate for each VFC pseudotyping virus/condition tested.

### Mice

Inbred Rocky Mountain White (IRW) strain mice were used for the isolation of primary CNS cultures. *mT/mG*, double-fluorescent Cre reporter mice (Muzumdar et al., 2007) Gt(ROSA)26Sortm4(ACTB-tdTomato,-EGFP)Luo on the129/SvJ background (Jackson Labs) were crossed with IRW mice (F1), and NPCs derived from the F1 mice, were used to facilitate the visualization of retroviral CNS targets, based upon the Cre-mediated excision of floxed *mTomato* and subsequent expression of membrane-associated green fluorescent protein (*mGFP*) driven by the chicken beta actin promoter. Brainbow 3.0 mice (strain H) (Livet et al., 2007) neuron-XFP Cre reporter mice were also used as a readout for Cre virus transduction of neurons. Brainbow 3.0 transgene mice were bred with IRW mice for a minimum of six generations prior to transplantation with virus-producing VFCs. Mice were bred and housed in the Comparative Medical Unit at the Northeast Ohio Medical University (NEOMED) in agreement with the American Association for the Accreditation of Laboratory Animal Care guidelines. All procedures were approved by the NEOMED Institutional Animal Care and Use Committee (IACUC).

### Fluorescence and Immunofluorescence Microscopy

*In vitro* assessment of astrocyte infection by ecotropic, amphotropic, or ecotropic-pseudotyped amphotropic viruses was performed in primary CNS cultures from IRW mice by double immunofluorescence staining. After 3 days of virus challenge, primary CNS cultures were fixed with 3.7% formaldehyde, incubated for 20 min with 3% BSA, followed by incubation with antibodies 83A25 (1:5 dilution of hybridoma supernatant), which recognized common epitopes to most MLV Envs (Evans et al., 1990) and anti-GFAP antibody (rabbit-anti GFAP, 1:1000 dilution, DAKO) for 1 h at RT. Cells were washed with PBS and incubated with secondary antibodies against Env and glial fibrillary acidic protein (GFAP), Alexa-fluor goat anti-mouse IgG 488 (1:1000) and Alexa fluor donkey anti-rabbit IgG 594 (1:1000) respectively. Cell were washed and fixed prior to confocal imaging analysis.

*In vivo* identification of mGFP cells was performed on 50 μm vibratome brain sections taken from mT/mG mice perfused with 4% paraformaldehyde in PBS. Tissues were permeabilized using 0.1% Triton X-100/PBS for 1 h at RT and then incubated in 3% BSA. In order to assure staining throughout the tissue, primary and secondary antibodies for the different cell type specific markers were incubated for about 18 h each, followed by Alexa Fluor streptavidin 647 for 5–6 h. Primary antibodies used included, rabbit-anti Iba-1 (1:500, WAKO) for microglia; rabbit-anti GFAP (1:1000, DAKO) for astrocytes; rabbit-anti carbonic anhydrase II (CA-II, 1:4000, Chemicon), rabbit-anti galactocerebroside (1:2000, Sigma) and mouse anti-2′,3′-cyclic nucleotide 3′-phosphodiesterase (CNPase, 1:500, Chemicon) for oligodendrocytes; rabbit-anti Olig2 (1:500, Chemicon) and rabbit polyclonal-anti PDGFR-α (1:300, Santa Cruz) for oligodendrocyte progenitor cells (OPCs) and oligodendrocytes (OLs); rabbit-anti NG2 (1:100, Chemicon); mouse-anti nestin (1:500, PharMingen) for neuronal progenitor cells; mouse-anti HuC/D (1:500, Invitrogen), mouse-anti-NeuN (1:100, Chemicon); rabbit-anti neurofilament-200 (NF-200, 1:2000, Sigma) and rabbit polyclonal-anti L1 (1:2000, Mellita Schachner) for neurons and lastly, rabbit-anti Factor VIII (BioGenex) for endothelial cells. Primary antibodies were detected using secondary or tertiary reagents coupled with Alexa 647 fluorescent dye (far red/near infrared; excitation maximum 650 nm, emission maximum 665 nm) excited by a He-Ne laser (633 nm) and imaged with a far red selective filter set to distinguish the signal from any mTomato emission. Although the mTmG mice constitutively express mTomato protein (orange-red), it is not excited by He-Ne laser line (excitation maximum 554 nm, emission maximum 581 nm) and its emission is very low in the near-infrared region of the filter set. Secondary antibodies included, Alexa Fluor donkey anti-rabbit 647 (1:1000, Invitrogen) and goat-anti mouse biotinylated (1:200, Southern Biotechnology Associates) and the tertiary reagent Alexa-fluor Streptavidin 647 (1:1000, Invitrogen). Images displayed in the figures for the signals collected were pseudo-colored red for alexa 647 for comparison to the GFP signals (pseudo-colored green).

Quantitative assessment of cell staining and morphology frequencies were performed by investigators who were blinded to the identity of the samples. Multiple group comparisons were performed using one-way analysis of variance with Tukey’s post-test as appropriate, while unpaired student *t*-tests were used for comparing two groups.

### Neural Stem Cell Transplantations

C17.2 NSCs or VFCs from confluent plates were detached using trypsin-EDTA, washed once with DM10N, and resuspended at a concentration of 5–9 × 10^7^ cells/mL in PBS containing 0.2% trypan blue. Cells were kept on ice until transplantation. Cell suspensions were injected into the brainstem (6 injections of 100 nl each) and lateral ventricles (2 injections of 100 nl each) of P0 cryoanesthetized mice using World Precision Instrument Micro 4 microsyringe pump controller. Pups were warmed on heating pad until activity levels returned to normal and then were returned to their parents. At 1, 2, and 3 weeks post-transplantation, mice were anesthesized under isoflurane and perfused with freshly prepared 4% paraformaldehyde in phosphate buffered saline. Brains were dissected from the calvarium, immersion-fixed in 4% PFA overnight and then stored in PBS at 4°C until either paraffin embedding for histological analysis or sectioning into thick sections (50 μm) for use in immunofluorescence or fluorescent protein localization by confocal microscopy outlined above.

## Results

### Ecotropic Pseudotyping by CasBrE Env Exacerbates Spongiform Neurodegeneration Mediated by the 4070A Amphotropic Virus

**FIGURE 1:**
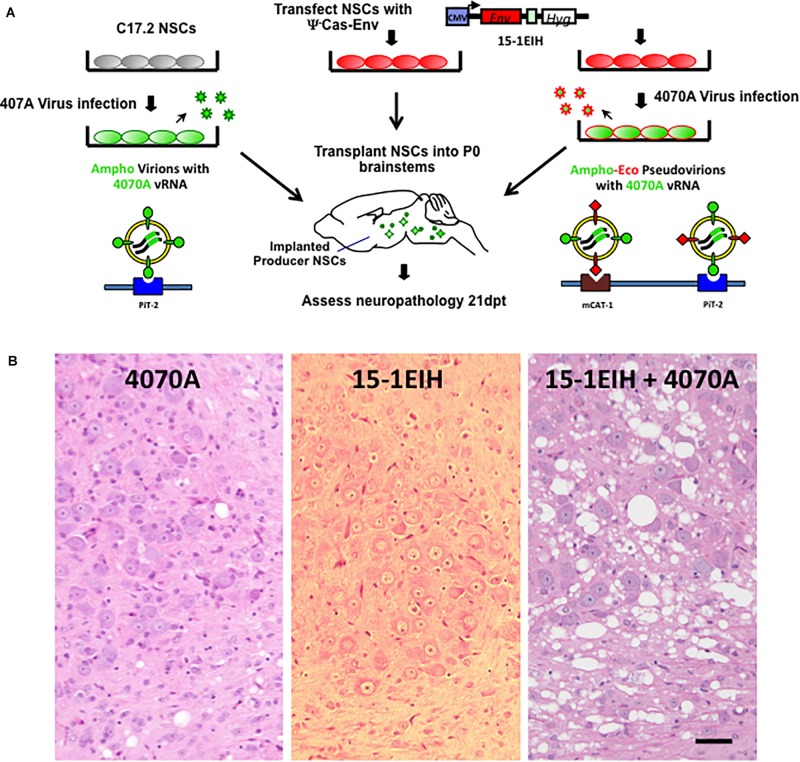
NSC-based amphotropic virus pseudotyping by ecotropic CasBrE Env dramatically exacerbates brainstem spongiform neurodegeneration. Panel **(A)** illustrates the experimental scheme for CNS pseudotyping of the 4070A virus. The diagram shows that C17.2 NSCs either with or without the 15-1EIH vector (to express the CasBrE ecotropic Env) were infected with the 4070A amphotropic virus and then transplanted into neonatal (P0) IRW mouse brainstems for subsequent histological evaluation. The nature of the virus particles produced are shown associated with the cell surface receptors they use to enter target cells, PiT-2 and mCAT-1. The 15-1EIH vector (Li et al., 2011) mRNA cannot be incorporated into viral particles due to the lack of a packaging sequence (psi minus; ψ^–^). CMV, cytomegalovirus promoter. Hyg, selection marker, hygromycin B phosphotransferase gene. Panel **(B)** shows representative hematoxylin and eosin staining of 10 μm paraffin sections from the brainstem, pontine reticularis for 4070A-NSCs (left), 15-1EIH-NSCs (middle) and 15-1EIH+4070A-NSCs (right) at 3 weeks post-transplantation in IRW mice. Note the number and size difference in vacuoles when 4070A virus is pseudotyped by CasBrE Env as previously reported in Li et al. (2011). Bar = 50 μm.

The 4070A amphotropic virus possesses a limited potential for inducing spongiosis in IRW and other mouse strains even when the virus is delivered within the CNS by engrafted C17.2 NSCs as outlined in Figure 1A (see also DesGroseillers et al., 1984; Traister and Lynch, 2002; Li et al., 2011). Figure 1B, left panel shows an example of the histology in the pons resulting from neonatal transplant of 4070A infected NSCs, 3 weeks post-injection. Limited but detectable vacuolation was noted in the pons and other engrafted brainstem regions. In contrast, transplantation of NSCs expressing CasBrE Env from a non-packaged expression vector (15-1EIH) failed to induce detectable neuropathological changes (Figure 1B, middle panel; Lynch et al., 1996). However, transplantation of NSCs with both 15-1EIH and 4070A to generate CasBrE Env pseudotyped 4070A virus, dramatically exacerbated 4070A spongiform neurodegeneration (Figure 1B, right panel; and Li et al., 2011). These results suggest that the ecotropic Env protein mediates 4070A virus entry into CNS cell types that express little or no PiT2, or express a version of the protein that limits 4070A Env interactions. Previous *in vivo* studies reported essentially equivalent infection of microglia, NG2 cells, OPCs, oligodendrocytes and endothelial cells by 4070A with and without ecotropic pseudotyping, suggesting that the ecotropic cellular infection was occurring in a cell type that was not detectable by normal immunohistochemical means (Li et al., 2011).

### Astrocyte Progenitors Are Efficiently Targeted by Ecotropic but Not Amphotropic Virus *ex vivo*

**FIGURE 2:**
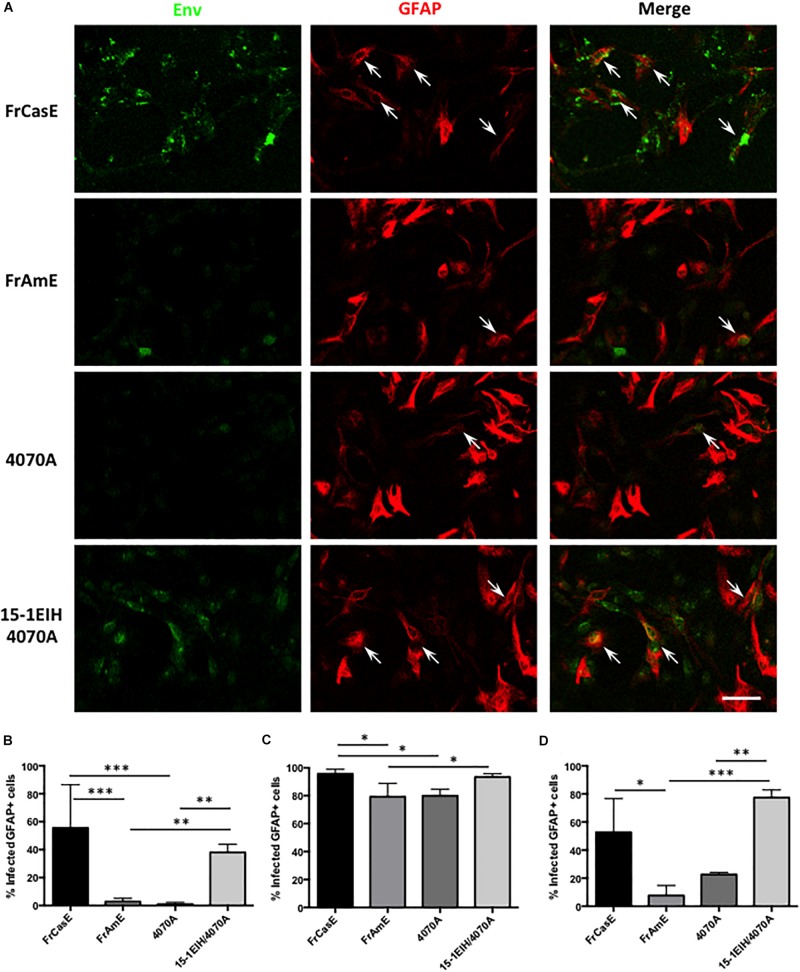
Astrocytes in acute primary CNS cultures are more efficiently infected by ecotropic versus amphotropic viruses. **(A)** Double immunostaining of P0 IRW primary CNS cultures for viral Env (monoclonal antibody 83A25′) (Evans et al., 1990), and astrocytes (anti-GFAP), 3 days post-exposure to FrCasE (ecotropic), FrAmE (isogenic amphotropic), 4070A (amphotropic), or 15-1EIH+4070A (ecotropic pseudotyped amphotropic) at a multiplicity of infection of 1. Note frequent colocalization of Env in GFAP+ cells (arrows) for FrCasE and 15-1EIH 4070A. Bar = 100 μm. Panel **(B)** shows a quantitative comparison of the percentage of GFAP+cells that were also Env+ for the groups displayed in **(A)**. Note the statistical differences between the ecotropic groups (FrCasE &15-1EIH/4070A) versus amphotropic groups (FrAmE &4070A). Panel **(C)** shows the percentage of GFAP+ cells that were Env+ when CNS cultures were grown for 10 days post-virus exposure. While a significant difference in susceptibility could be observed for some ecotropic versus amphotropic infections, astrocyte restriction of amphotropic virus appeared to wane with time in culture as illustrated by 4070A versus 15-1EIH/4070A. Panel **(D)** shows a comparison of primary astrocyte susceptibility to ecotropic versus amphotropic virus when the CNS cultures were grown for 7 days prior to virus exposure, and then assayed at 3 days post-exposure. Note significant differences between amphotropic and pseudotyped amphotropic viruses, but with some indication that culture conditions can affect astrocyte amphotropic susceptibility. Error bars = standard deviation. ^∗^*P* < 0.05; ^∗∗^*P* < 0.01, ^∗∗∗^*P* < 0.001.

Previous work from our laboratory has shown that mixed glial cultures from IRW mice could be readily infected by ecotropic MLVs, with Env expression detectable in astrocytes, oligodendrocytes, OPCs and microglia (Lynch et al., 1994; Li et al., 2016). To examine whether glial progenitors might be differentially targeted by ecotropic versus amphotropic virus, P0 neonatal brain tissue from IRW mice was freshly dissociated and then immediately exposed to ecotropic (FrCasE), amphotropic (FrAmE and 4070A), or CasBrE Env-pseudotyped amphotropic (15-1EIH-4070A) viruses at an MOI of 1 and grown as primary CNS culture monolayers in the presence of serum to favor astrocyte differentiation (cf. Foo et al., 2011). At various times after virus exposure, the cultured cells were immunostained for Env expression (green) and the astroglial specific marker GFAP (red), as illustrated in Figure 2A. Note that Env and GFAP colocalization were readily detected in cells exposed to ecotropic (FrCasE), and ecotropic-pseudotyped amphotropic viruses (15-1EIH-4070A) but more limited colocalization was seen with either 4070A or FrAmE, when assessed 3 days post-initial virus exposure. Quantitation of astrocyte infection showed highly significant differences between the ecotropic and amphotropic virus-exposed groups (Figure 2B). Interestingly, if mixed glial cultures were grown for 10 days post-virus exposure, the percentage of GFAP+ cells expressing Env was much higher for all virus groups (Figure 2C), suggesting that resistance to amphotropic virus infection wanes with time in culture and/or cell-to-cell spread. Similarly, astrocytes/progenitors that were in culture for 7-days prior to virus exposure were more permissive for amphotropic infection (Figure 2D). These results suggest that the glial progenitor cell susceptibility changes with time in culture. Nonetheless, the same general trend of astrocyte restriction to amphotropic infection appeared in all three experimental paradigms, supporting the idea that astrocytes may represent a CNS cell type that is differentially targeted by ecotropic and amphotropic viruses *in vivo*.

### Neural Progenitor Cells Are Resistant to Amphotropic but Not Ecotropic Virus Infection

We recently demonstrated that primary neural progenitor cells (NPCs) grown as neural spheres (NPHs) could be infected with neurovirulent and non-neurovirulent ecotropic viruses *ex vivo* followed by transplantation into developing brains to assess NPC survival, integration and glial fate determination (Li et al., 2016). However, this study did not explore whether IRW NPHs and the associated culture conditions would facilitate infection by amphotropic viruses. C17.2 NSCs, by comparison, were previously shown to be susceptible to either ecotropic or amphotropic viruses *in vitro* (Traister and Lynch, 2002). Moreover, these cells could engraft, migrate extensively, express Env, and efficiently disseminate virus to surrounding cells upon transplantation within the developing brain (Lynch et al., 1996, 1999; Traister and Lynch, 2002; Li et al., 2011), although their capacity to integrate as differentiated glia remains an open question. Nonetheless, transplanted 4070A-infected C17.2 NSCs could not bypass the restriction to amphotropic neurovirulence in IRW mice unless pseudotyped by an ecotropic Env (Figure 1 and Li et al., 2011). We therefore wanted to examine whether the *in vivo* amphotropic restriction would extend to NPCs cultured as NPHs when placed in direct contact with C17.2 NSCs.

**FIGURE 3:**
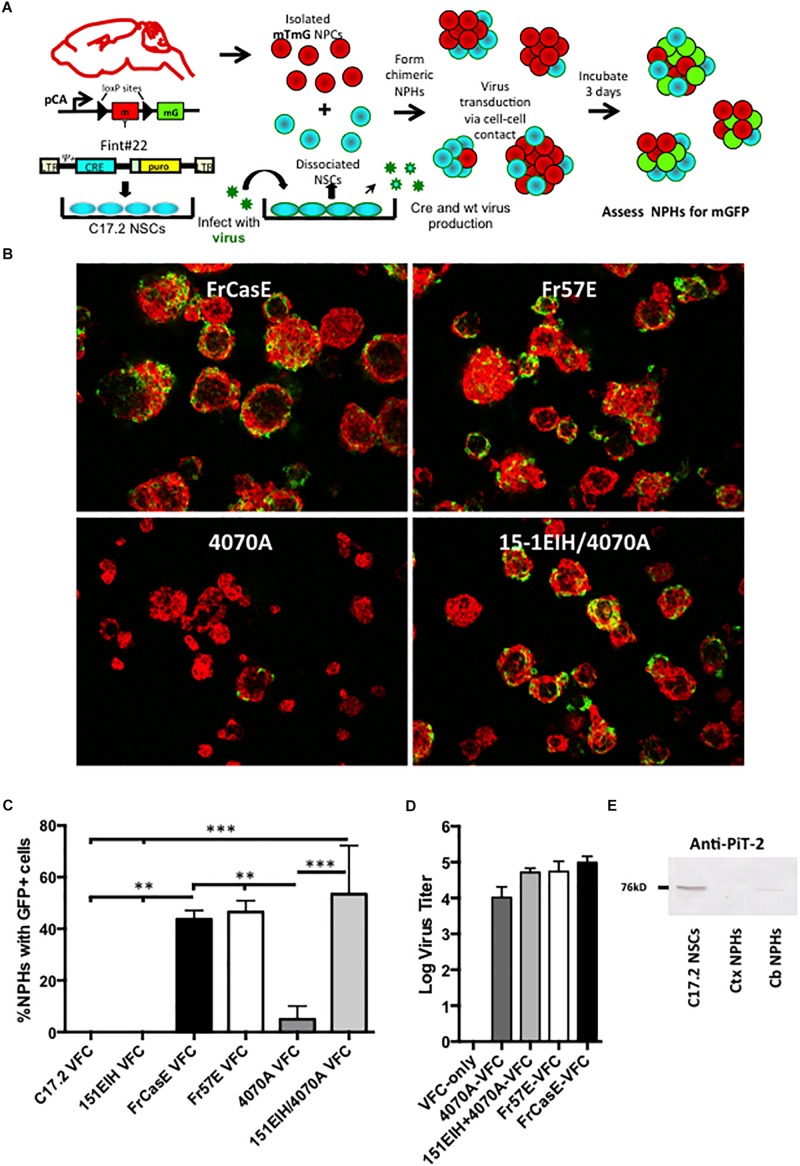
Neural progenitor cells grown as neural spheres resist cell–cell infection from amphotropic but not ecotropic virus. Panel **(A)** illustrates how neural progenitor cells were evaluated for their permissiveness for ecotropic versus amphotropic MLV infection in a cell–cell contact paradigm. Specifically, primary neural progenitors isolated from the cortices of mTmG transgenic Cre-reporter mice were combined with C17.2 NSCs transduced with a packagable Cre retroviral vector (FINT#22; Westerman and Leboulch, 1996; called VFCs) and infected with FrCase or Fr57E (ecotropic) or 4070A (amphotropic) or CasBrE-pseudotyped 4070A (15-1EIH+4070A), and grown as chimeric neurospheres (NPHs). After 3 days NPHs were assayed for GFP expression, as an indicator of virus infection by ecotropic or amphotropic Cre vector pseudotypes. **(B)** Examples of merged low power images of chimeric NPC/VFC neural spheres expressing mTomato (red) and mGFP (green) at 3 days. Note abundant mGFP expression in NPHs with made with VFCs producing ecotropic pseudotyped viruses but infrequent mGFP+ NPHs with 4070A pseudotyping. Bar = 50 μm. **(C)** Quantitative comparison of the percentage of NPHs with mGFP+ expressing cells when chimeric NPHs were assembled with mTmG NPCs and either C17.2-VFCs, 15-1EIH-C17.2-VFCs, FrCasE-C17.2 VFCs, Fr57E-C17.2 VFCs, 4070A-C17.2 VFCs or 151EIH+4070A-C17.2 VFCs. Error bar = standard deviation. ^∗^*P* < 0.05; ^∗∗^*P* < 0.01, ^∗∗∗^*P* < 0.001. Panel **(D)** shows the ecotropic and amphotropic virus titers released from the C17.2 VFCs used for the chimeric neural sphere assay as an indicator of the potential for pseudovirion release from these cells. **(E)** Western blot for PiT-2 expression in total cell extracts from C17.2 NSCs (which are susceptible to amphotropic virus infection) or NPH cultures derived from the neonatal cortex (Ctx) or neonatal cerebellum (Cb). Note the detection of PiT-2 in C17.2 cells, with low level expression in NPCs from the cerebellum, but undetectable expression in those from the cortex.

To answer this question we developed a methodology for monitoring retrovirus transduction that would be largely independent of viral structural protein synthesis in the target cell, and thus, bypass any virus suppression that might occur post-virus integration. As outlined in Figure 3A, this method employs NCS-based viral pseudotyping of a Cre recombinase encoding retroviral vector, FINT#22 (Westerman and Leboulch, 1996), used in combination with NPCs derived from the global double fluorescent Cre reporter mouse (mTmG), which constitutively express the membrane targeted Tomato (mT) fluorescent protein from the chicken beta-actin promoter. Upon *mT* gene excision by Cre recombinase, the actin promoter drives constitutive membrane green fluorescent protein (mGFP) expression (Muzumdar et al., 2007). Infection of mTmG targets by virions containing the Cre vector would be expected to induce transgene recombination and mGFP expression after reverse transcription, integration, and expression of the Cre recombinase. To test this strategy, NPCs derived from an F1 IRW × mTmG mating were grown as neural spheres (NPHs), dissociated into single cells, and then mixed with Cre vector (FINT#22) infected/selected C17.2 NSCs (C17.2 VFCs) or 15-1EIH C17.2 VFCs, with and without infection with FrCasE, Fr57E, or 4070A. Chimeric NPHs were assessed after 3 days in culture for mGFP expression as an indicator of vector spread and Cre expression.

As shown in Figure 3B, NPHs with mGFP+ NPCs were readily observed when chimeras were made with VFCs infected with the ecotropic viruses FrCasE, Fr57E, or engineered with CasBrE Env (15-1EIH) to pseudotype the 4070A virus. In contrast, mGFP+ NPHs were infrequently observed with 4070A-VFCs without ecotropic pseudotyping. Quantitative comparison of the percentage of NPHs with mGFP+ cells observed for the different virus/Env combinations showed highly significant restriction to amphotropic infection (Figure 3C) by the Cre-mediated neural sphere assay. Importantly, chimeras made with C17.2 VFCs (FINT#22 only) or VFCs encoding the CasBrE Env alone (15-1EIH), failed to result in any detectable mGFP+ NPHs, consistent with the need for the Cre vector to be transduced by retrovirus pseudotyping in order to generate mGFP+ cells. Pseudotyping virus titers for the VFCs used are shown in Figure 3D.

To assess whether NPC resistance to 4070A infection might be due to the lack of expression of the amphotropic receptor, PiT2, Western blots were carried out on whole cell extracts of cultured NPHs derived from P0 cortex or cerebellum and compared to C17.2 NSCs (Figure 3E). PiT-2 immuno-reactivity was observed for C17.2 cells but was not noted for NPHs derived from the cortex. Interestingly, NPHs derived from P0 cerebellum showed a very low but detectable expression level of PiT-2 immuno-reactivity, although a detailed analysis of cerebellar NPC susceptibility to 4070A infection was not undertaken. Whether these results reflect region-specific PiT-2 expression, or reflect different neural developmental expression was not clear. Whether the changing susceptibility of the glia with time in culture may also reflect different receptor expression levels was not investigated. Nonetheless, these results indicate that primary NPCs are largely refractory to infection from amphotropic virus transmitted by cell–cell contact.

### *In vivo* NSC-Based Virus Pseudotyping Identifies CNS Target Cells With Subdetectable Env Protein Expression

**FIGURE 4:**
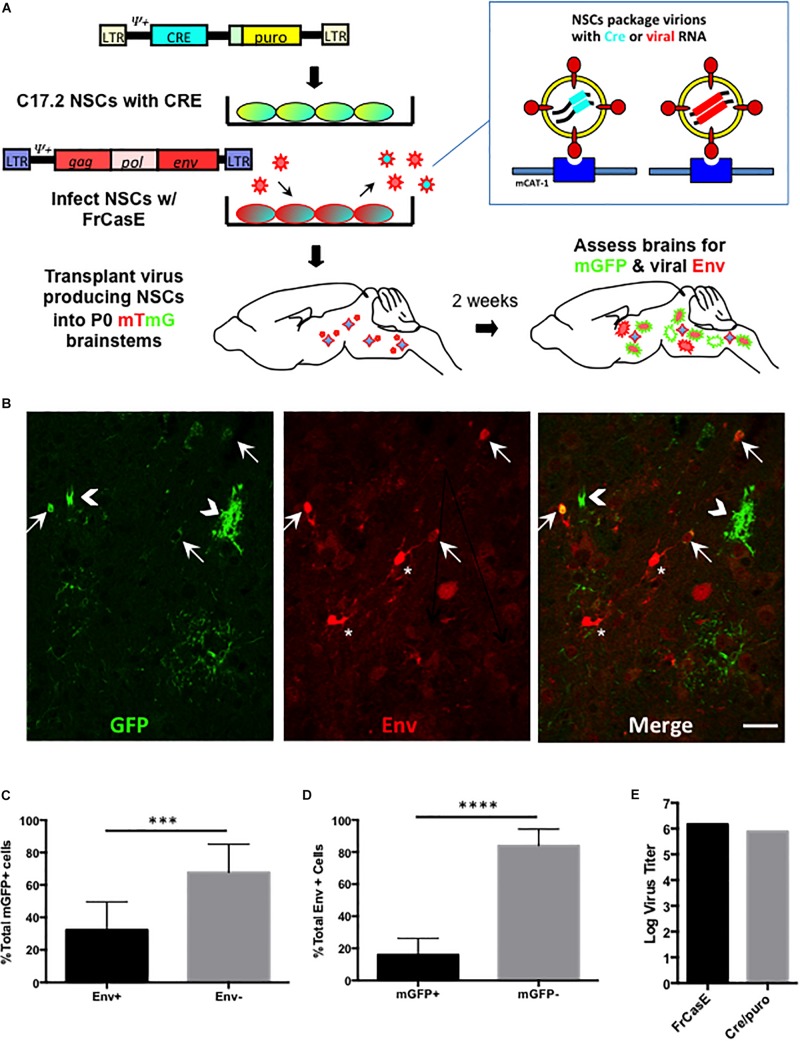
FrCasE pseudotyping of Cre in the CNS identifies cellular targets with subdetectable viral Env protein expression. Panel **(A)** illustrates the strategy employed for assessing whether there are ecotropic virus target cells that suppress virus expression to subdectable levels. As outlined in Figure 3, this method employs mTmG the transgenic reporter mouse, and uses virus pseudotyping of the Cre retroviral vector as a sensor for infection in parallel with immunodetection of viral protein expression *in situ*. Panel **(B)** shows a representative brainstem section indicating that NSC-mediated FrCasE pseudotyping induces mGFP expression in mTmG × IRW F1 mice examined 14 days post-transplantation (green, left panel). Immunostaining (red, middle panel) with 697, a CasBrE-specific monoclonal antibody identifies cells within the same section that express viral Env. The merged image (right panel) shows the existence of cells that express GFP and Env (arrows), cells that express GFP only (chevrons) and cells that express Env alone (asterisks). Bar = 20 μm. Panel **(C)** shows quantitative assessment of brainstem regions for Env expression in GFP+ cells examined in five separate mice indicating that approximately two-thirds of GFP+ cells failed to express coincident Env expression. **(D)** Env+ cells in the brainstem were assessed for coincident GFP expression as indirect measures of (1) NSC engraftment and (2) the extent to which superinfection interference might restrict Cre virus mediated conversion of mTomato to mGFP. *N* = 4 mice. Error bars represent standard deviation. Comparison by two tailed unpaired Student’s *t-*test. ^∗∗∗^*P* < 0.001; ^∗∗∗∗^*P* < 0.0001. **(E)** Virus titers for the pseudotyping virus, FrCasE and Cre/puro encoding virus run in parallel on the same supernatant. FrCasE titer is FFU/ml whereas Cre/puro titers are CFU/ml.

Our *in vitro* results above suggest that neural and astrocyte progenitors are refractory to amphotropic but not ecotropic infections. To address whether this differential tropism occurs in the developing brain, and examine whether certain ecotropic targets suppress virus expression, we extended the NSC-based mTmG reporter detection strategy to the *in vivo* setting. As outlined in Figure 4A, C17.2 VFCs were infected with FrCasE, transplanted into F1 IRW × mTmG neonatal brains and then examined 2 weeks later for the presence of mGFP and Env expressing cells. Figure 4B shows a representative brainstem section from a mouse transplanted with FrCasE-infected VFCs, immunostained for the CasBrE Env using the mouse monoclonal antibody 697. Note cells expressing GFP without coincident Env expression (chevrons), cells expressing GFP and Env (arrows), and cells expressing Env without GFP (asterisks). Transplantation of uninfected VFCs failed to show detectable mGFP expression (not shown), consistent with the *in vitro* experiments indicating the need for a pseudotyping virus to transfer the Cre vector and generate recombinase activity. Quantitation of Env+, GFP+ coincidence in FrCasE-VFC transplanted mice showed that approximately one third of the GFP+ cells in the brainstem were also positive for Env expression (Figure 4C; *N* = 5). This result suggests that immunostaining for Env significantly underestimates the FrCasE CNS target population, while supporting the idea that certain infected neural cells have the capacity to suppress virus expression (Li et al., 2016).

A similar quantitative analysis of the Env+ cells in the FrCasE-VFC transplanted mice indicated that only about 20% of the total were GFP+ (Figure 4D). The large population of Env + GFP_neg_ cells likely represented the engrafted FrCasE-VFCs themselves. In addition, host cells that were initially infected by FrCasE virus without coincident FINT#22 transduction would be expected to establish superinfection interference, and thus prevent subsequent Cre encoding virions from entering and infecting these cells. By contrast, cells expressing GFP that did not co-express Env, likely represented viral target cells that suppressed FrCasE expression, since transduction by FrCasE pseudotyped Cre only encoding virions, would not establish any superinfection interference until infected by a FrCasE encoding virus.

### CNS Viral Cre Pseudotyping Indicates That Astrocytes Constitute a Major Unrecognized CasBrE Virus Target in the Developing CNS

**FIGURE 5:**
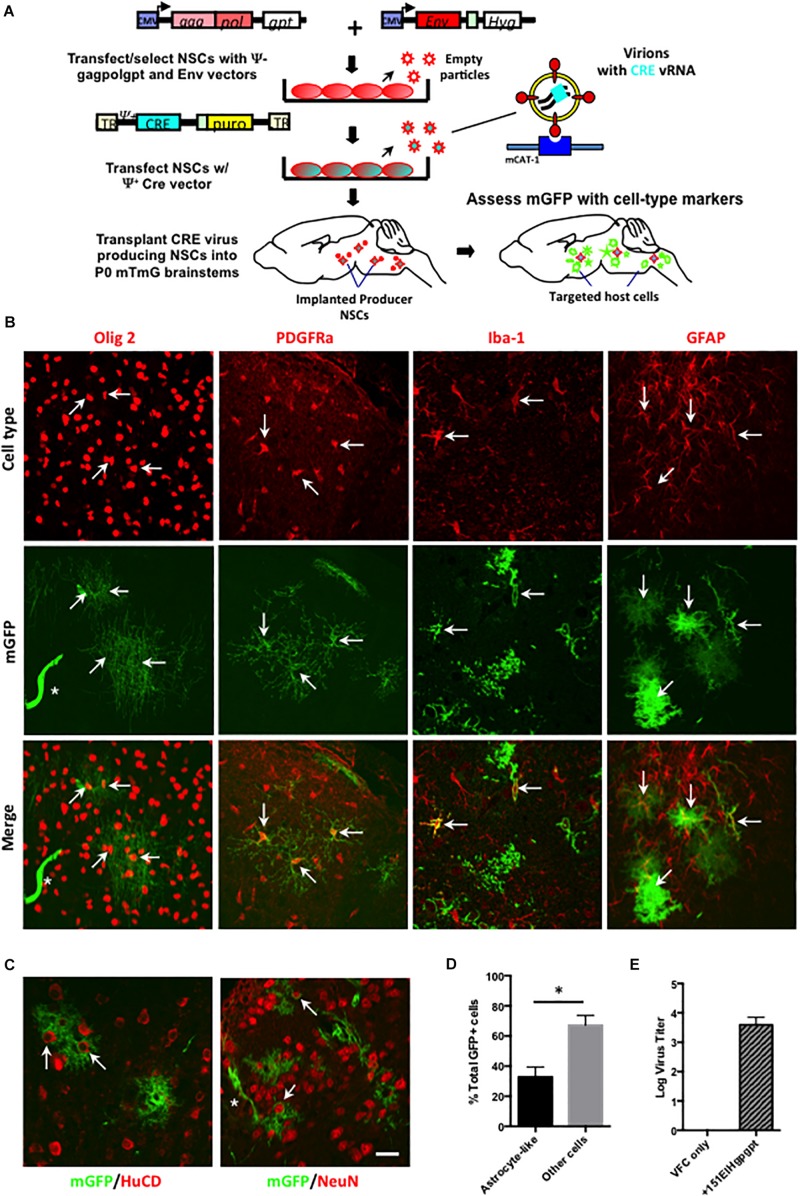
Packaging/producer NSC-mediated Cre pseudotyping identifies astrocytes as a major unrecognized CasBrE virus target in the developing CNS. **(A)** Packaging/producer NSCs were generated by transfecting the 15-1EIH and Fint#22 plasmids into gag-polgpt C17.2 NSCs (Li et al., 2011) followed by selection. Cre transducing cells were transplanted into P0 mTmG × IRW F1 brainstems followed by examination for mGFP expression and cell type specific markers at 2 weeks. Panel **(B)** shows representative examples of cell-type specific immunostaining (top row, red) coincident with Cre-induced mGFP expression (middle row). The merged images (bottom row) for oligodendrocytes (anti-Olig2; first column), oligodendrocyte progenitor cells (OPCs; anti-PDGFRa; second column), microglia (anti-Iba-1; third column), and astrocytes (anti-GFAP; fourth column). Examples of cells showing coincident marker and GFP labeling are indicated by arrows, determined based on juxtaposition of cell processes, cytoskeletons, nuclei, and membrane morphologies observed in confocal z-stacks. GFP expression was also noted in cells consistent with vascular endothelia based on cellular morphology (asterisks). Bar = 20 μm. Panel **(C)** shows mGFP expression juxtaposed to immunostaining for neuronal markers HuC/D (left panel) and NeuN (right panel). Note that although GFP expression could be seen closely associated with neuronal cell bodies (arrows), confocal z-stack assessment did not support colocalization of GFP within brainstem neurons in any animals examined. Staining instead appears to reflect GFP expression in closely associated astrocytes. Bar = 20 μm. **(D)** Quantitation of GFP+ cells exhibiting protoplasmic astrocyte-like morphologies versus all other cell morphologies, expressed as a percentage of the total GFP+ cells. Error bars represent standard deviation. Group comparisons were carried out by unpaired Student’s *t-*tests. ^∗^*P* < 0.05. *N* = 4 mice. Panel **(E)** shows the Cre/puro virus titer obtained for VFC alone or engineered with the packaging plasmids gagpolgpt and 151EIH in CFU/ml.

To identify the CNS cell types that were being targeted by ecotropic viruses, but avoid the superinfection interference confound associated with replication competent virus, the Cre pseudotyping strategy was modified (Figure 5A) to employ packaging producer-VFCs possessing CasBrE Env and gag-pol viral protein expression vectors (151EIHgpgpt-VFCs) to package only the psi+ (ψ+) Cre vector (FINT#22). Assessment of Cre/puro encoding virus titers from the packaging VFCs in culture (Figure 5E) indicated that it was much lower than that noted for VFCs infected with replication competent FrCasE (Figure 4). Nevertheless, as shown in Figure 5B, transplantation of the packaging/producer 15-1EIHgpgpt-VFCs into P0 mTmG × IRW F1 mice followed by CNS assessment at 2 weeks post-transplant demonstrated the appearance of many GFP+ cells with a variety of distinctive morphologies. Vascular cells, endothelia and pericytes were identified morphologically by their tubular shapes with limited processes in close association with blood vessels (white asterisks). Other target cell types were identified by both their morphologies and by mGFP colocalization with cell type-specific markers, including, GFAP for astrocytes, Olig2 for myelinating and premyelinating oligodendrocytes, platelet-derived growth factor receptor alpha (PDGFRa) and NG2 (not shown) for polydendrocytes/oligodendrocyte progenitor cells (OPCs), and Iba-1 for microglia. Examples of mGFP colocalization with each of these glial markers was observed (Figure 5B, arrows), which included astrocytes possessing both protoplasmic (bushy/cottony morphology) and fibrous (straighter, fewer, and longer processes) phenotypes. Astrocyte targeting was of particular interest since this cell type has rarely been shown to express viral protein in CasBrE and FrCasE infected mice in brain regions undergoing neurodegeneration (Lynch et al., 1991; Gravel et al., 1993).

We have previously reported that neurons in brain regions undergoing neurodegeneration show evidence for virus binding and entry into the cytoplasm but failure to express viral proteins (Lynch et al., 1991), consistent with other reports suggesting that neurovirulent virus infection of susceptible neurons may be abortive (Sharpe et al., 1990). However, utilizing the Cre pseudotyping strategy, no mGFP expression was observed in cells expressing the neuronal markers HuC/D or NeuN (Figure 5C). These findings were consistent with a failure of FINT#22 vector to integrate and be expressed in brainstem neurons, if/when virus entry occurred. Importantly, neurons appeared to be in intimate contact with mGFP+ cells with morphologies characteristic of protoplasmic astrocytes (Figure 5C, arrows), that showed them wrapping their processes around the neuron cell body in a way consistent with the early camera lucida drawings by Cajal (Garcia-Marin et al., 2007). To further exclude the possibility of neuronal infection, 15-1EIHgpgpt-VFCs were also transplanted into neonatal Brainbow 3.0 Cre reporter mice (strain H), where Cre-mediated conversion of fluorescent protein expression is restricted to neurons (Livet et al., 2007). No fluorescent protein conversion (cyan fluorescent protein or yellow fluorescent protein) was observed in transplanted brainbow transgenic animals (*N* = 4) that had been bred onto the IRW background through the F6 generation, when examined 4 weeks post-transplant (not shown). However, it should be pointed out that Brainbow 3.0 strain H mice were not a robust readout system in young mice since the levels of constitutive RFP expression in brainstem neurons was quite low and was variable between neurons. Nonetheless, the absence of detectable changes was consistent with the mTmG results.

Previous studies investigating CasBrE neuropathogenesis had consistently reported very low levels of astrocyte infection based on viral protein expression as the readout (Lynch et al., 1991; Gravel et al., 1993; Li et al., 2011, 2014). In contrast, mGFP+ astrocytes were readily observed in the current analysis, and given that protoplasmic astrocyte morphologies were easily distinguishable from other cell types based on their characteristic bushy/cottony phenotype, we quantified their incidence among GFP+ CNS targets to gain a perspective on the frequency of their infection (Figure 5D). The results showed that protoplasmic astrocyte-like cells represented approximately one third of all the mGFP+ cells, a number dramatically higher than previous reports of 0–0.3% for astrocytes (Gravel et al., 1993; Li et al., 2011). Thus, the current results using the mTmG reporter mice suggest that astrocytes represent a major CNS target of the CasBrE virus, despite their failure to express detectable levels of viral protein. This finding is consistent with our recent report indicating that glial progenitor cells down-regulate virus protein expression upon astrocyte differentiation and tissue integration (Li et al., 2016).

### CNS Cre Pseudotyping Reveals Differential Cellular Targeting by Amphotropic Versus Ecotropic Viruses

**FIGURE 6:**
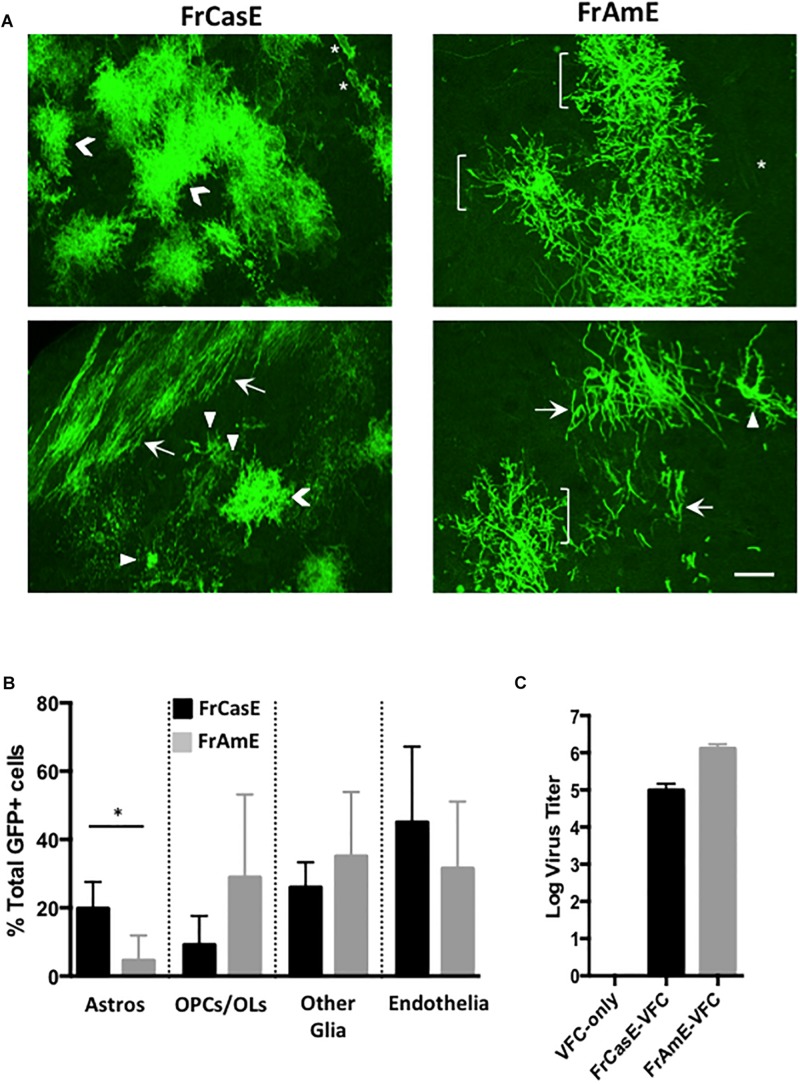
FrCasE- versus FrAmE-Cre pseudotyping indicates that amphotropic astrocyte infection is inefficient *in vivo*. **(A)** Two representative examples of flattened confocal stacks are shown of mGFP+ cells within the brainstems of mice transplanted with FrCasE- or FrAmE-C17.2 VFCs (Cre vector+) at 14 days post-injection. The GFP+ cells observed were categorized morphologically into one of four groups: (1) protoplasmic astrocytes (Astros; chevrons); (2) premyelinating oligodendrocytes (OPCs; brackets) and myelinating oligodendrocytes (OLs; arrows); (3) other glia (microglia, NG2 glia, fibrous astrocytes; arrowheads); and endothelia/vascular cells (asterisks). Bar = 20 μm. **(B)** Quantitative comparison of the percent of total GFP+ cells in each morphological group for FrCasE versus FrAmE pseudotyping viruses in engrafted brainstem sections collected from VFC transplanted mice (FrCasE, *N* = 3; FrAmE, *N* = 4). Group comparisons between FrCasE and FrAmE were carried out by unpaired Student’s *t-*tests. Error bars represent standard deviation. Only Astros showed a significant difference between viruses. ^∗^*P* < 0.05. **(C)** Comparison of the titers of the pseudotyping viruses released from the cultured VFCs employed in the transplantation analysis.

In order to examine whether astrocytes and/or their progenitors constituted cell types that were preferentially targeted by ecotropic but not amphotropic viruses, we employed C17.2 VFCs infected with either FrCasE or FrAmE viruses to pseudotype Cre upon transplantation into mTmG × IRW F1 mice. FrAmE is isogenic to FrCasE except that it contains the amphotropic 4070A *env* gene (Traister and Lynch, 2002), and thus, any tropism differences would be solely attributable to the Env protein difference rather than any minor differences in other structural protein genes or LTRs. Moreover, it has been previously established that FrAmE virus does not induce acute progressive neuropathology upon dissemination within the brain by NSCs (Traister and Lynch, 2002). The assessment of cell types expressing mGFP (Figure 6A) was carried out blinded by examining confocal stacks of thick sections taken from the midbrain through the hindbrain of mice transplanted with C17.2 VFCs + FrCasE or FrAmE (*N* = 3 and 4 respectively per group). Cells were binned into cell-subtype categories based on their distinctive morphologies that included protoplasmic astrocytes (Astros; chevrons); OPCs and myelinating OLs (brackets and arrows respectively); endothelia (asterisks); and other glia (arrowheads) that included microglia, NG2 glia and fibrous astrocytes, cells that were morphologically difficult to distinguish from one another. Quantitative comparison between FrCasE and FrAmE targeting frequency showed no statistical differences between OPCs, myelinating and non-myelinating OLs (OPCs/OLs); other glia; and vascular endothelial cells when compared by unpaired *t*-tests (Figure 6B). In contrast, there was a fourfold higher incidence in the targeting of cells morphologically consistent with protoplasmic astrocytes in mice transplanted with VFCs pseudotyping Cre by FrCasE versus FrAmE. Analysis of the VFCs prior to transplantation indicated that those infected with FrAmE appeared to produce higher levels of pseudotyping virus than those infected with FrCasE (Figure 6C), however, it is unknown whether this would affect the *in vivo* transduction efficiency of Cre.

## Discussion

In this study, we were trying understand how ecotropic Env pseudotyping of minimally neurovirulent amphotropic retroviruses could induce severe spongiform neurodegeneration without the appearance of overt virus expression in a new or more abundantly targeted neural cell population. The presumption was that neurodegeneration was exacerbated because the ecotropic Env facilitated amphotropic virus entry and expression in a CNS cell population which failed to express the amphotropic receptor. Unfortunately, identifying that cell population was not possible using the standard tools used for defining CNS viral targeting, namely the detection of viral nucleic acids and/or proteins within that cell. Multiple cellular processes exist that can repress mobile genetic elements including endogenous and exogenous retroviruses, such as histone acetylation, DNA methylation, cytidine deamination, and zinc finger protein binding (cf. Matsui et al., 2010; Narasipura et al., 2014; Fasching et al., 2015; Wolf et al., 2015). Therefore, to appropriately address the neurotropism issue, we needed to develop new or alternate approaches to determine whether virus repression in the CNS was in fact occurring with MLVs.

Support for the idea that certain neural cells might suppress virus expression included studies on the rapidly neuropathogenic MLVs, where astrocyte expression of viral nucleic acid and viral Env protein was undetectable, but was readily detectable in microglia, endothelia, NG2/OPCs, and neural progenitors in infected brain sections (Baszler and Zachary, 1990, 1991; Morey and Wiley, 1990; Lynch et al., 1991; Gravel et al., 1993; Clase et al., 2006; Li et al., 2014). In contrast, culture experiments on primary glia showed productive infection of astrocytes (Lynch et al., 1994; Liu et al., 2004; Qiang et al., 2006). Given that astrocytes could be targeted by ecotropic viruses and were capable of expressing viral gene products (at least in culture), we translated this finding into a culture strategy to demonstrate that amphotropic virus entry was restricted in these cells, but could be bypassed by ecotropic pseudotyping. However, the robustness of the amphotropic restriction appeared dependent upon culture conditions and time *in vitro*. Because we previously showed that NPCs could be infected with ecotropic MLVs and maintained in culture as NPHs (Li et al., 2016), we extended our culture assessment to NPCs using a method that could be translated into the *in vivo* setting, even in the face of non-productive infection. These experiments showed that NPCs were also resistant to amphotropic infection. Comparative Western immunoblotting assessment indicated that restriction in these cells was likely due to limited expression of the amphotropic receptor PiT-2, rather than either a failure to transport it to the cell surface, or expressing a form of the protein that did not interact with the 4070A Env. The degree of amphotropic resistance in NPCs was especially striking given that in the chimeric NPH assay infection occurs primarily through cell–cell contact, a process known to dramatically elevate virus transduction efficiency (cf. Lynch et al., 1994).

In comparing the chimeric NPH culture analysis with the results obtained *in vivo*, using the NSC-based FrAmE Cre pseudotyping, the latter assay demonstrated that cells consistent with pre-myelinating and myelinating OLs are CNS amphotropic targets. The results suggest that 4070A targets a more committed oligodendrocyte progenitor cells, whereas earlier progenitors (NPCs) or those committed to astrocytes are more resistant. Given the potential for *in vitro* direction of NPC’s into differentiating brain organoids, the NPH systems could be further exploited to provide a more precise definition of the developmental stage at which neural progenitor cells become susceptible to amphotropic infection. Interestingly, the findings obtained here with Cre-mediated detection of virus targets are in contrast to prior reports documenting the distribution of PiT-2 in mouse brains. In these studies neither OLs nor their progenitors were identified using immuno-staining methods (Inden et al., 2013, 2016). Instead, PiT-2 was expressed in multiple neuron populations, vascular cells, and astrocytes. However, it is important to point out that the immunostaining studies used adult C57B6 mice versus the P14-21 day old IRWx129 strain animals used for the present studies. Moreover, it is not clear what cell surface receptor density is required for efficient virus transduction, so the detection limit of the differing methods become a potential confound. Finally, the viral targets identified with Cre pseudotyping reported here are restricted to dividing cells in order to facilitate vector integration, Cre expression, recombination and ultimately mGFP expression. Nonetheless, reporter readouts like that described here have the potential to reveal the presence of proteins in low abundance whose presence has dramatic biological implications.

It is important to highlight the Cre recombinase strategy we used to overcome the limitation of detectable virus protein expression levels. In this indirect method, the cellular substrate is limited to a single floxed mTomato sequence in F1 mice, and thus very little Cre recombinase was needed to mediate the switch from mTomato to mGFP expression. Because the readout gene is under control of the constitutive beta actin promoter, no virus life cycle events beyond expression of the integrated provirus were required for target detection. Because of the sensitivity of the readout, it is important to note that transplantation of NSCs expressing the Cre vector only, did not result in any mGFP conversion indicating that release of Cre recombinase from dying cells or through cell–cell fusion was not occurring. Equally notable was the fact that we did not detect evidence for infection of brainstem neurons, despite our previous observation of viral particle entry into these non-mitotic cells (Li et al., 2011). The lack of neuronal mGFP expression strongly argues that the Cre virus must be reverse transcribed and integrated during cell division in order for the mTomato to mGFP transition to occur. Going forward, the ability to visualize cryptically infected cells in live slices and intact functioning brains will allow for a detailed spatial and physiological analysis of their properties and the effects on neighboring neurons to elucidate their role in the neurodegenerative process. Moreover, this tool will allow for flow cytometric sorting of dissociated tissue for detailed single cell analysis and provide for understanding cytoarchitectural context for the developing synaptic vacuolar pathology.

Remarkably, the Cre reporter system indicated that approximately two-thirds of FrCasE viral CNS targets suppress virus expression to subdetectable levels. Critically, these experiments also showed that half of those virally suppressed cells were protoplasmic astrocytes based on morphological criteria. Previously, these astrocytes were largely discounted as important in neuropathogenesis due to the lack of detectable infection (Lynch et al., 1991; Gravel et al., 1993). Given the Cre-mediated detection of this cell type with FrCasE pseudotyping, and our failure to observe astrocyte expression of amphotropic Env proteins after ecotropic pseudotyping (Munk et al., 1998; Li et al., 2011), suggests that general retroviral suppression mechanisms are particularly active in this CNS cell type. In this regard, epigenetic retrovirus suppression in astrocytes has been widely reported for HIV infections in the human CNS (cf. Narasipura et al., 2014). And, while CNS targets undergoing viral protein suppression constitute persistent CNS viral reservoirs, it remains controversial whether neural cell infection, without detectable virus expression could play a causative role in neurodegeneration.

The paradigm investigated here, wherein viral pseudotyping facilitates severe neurodegenerative changes provides a strong argument that the apparently silent/suppressed astrocyte infection is a critical requirement for retrovirus-induced neurodegeneration. This finding is antithetical to the prevailing thought in diseases of abnormal protein-induced neurodegeneration, namely, that the abnormal neurotoxic proteins needs to accumulate to significant levels within the CNS in order to cause neuropathologic sequelae. Whether the productive infection of one or more other CNS cells such as NPCs, microglia and endothelia are also are required to cause spongiosis, remains to be address. The idea that the suppressive process itself might play a contributing role in the neurodegenerative process needs to be further explored, especially given our prior findings that ecotropic infection of NPCs could interfere with their differentiation into OLs upon transplantation into the developing brain (Li et al., 2016). In considering other possible underlying mechanism associated with non-productive infection, insertional mutagenesis has been previously explored (Portis et al., 1990; Lynch et al., 1991; Askovic et al., 2000). Studies comparing the isogenic ecotropic MLVs FrCasE and Friend, indicated that while both are highly neuroinvasive, and have indistinguishable neurotropisms, their dramatic differences in neurovirulence appear to preclude this mechanism (Portis et al., 1990; Lynch et al., 1991; Askovic et al., 2000; Li et al., 2014). Caution must be exercised in this interpretation, since glial infection by the non-neurovirulent Friend virus Fr57E, also induces minor neurophysiological changes in rebound neurons (Li et al., 2014) and both FrCasE and Fr57E viruses can alter the capacity of NPCs to become mature OLs after CNS transplantation (Li et al., 2016). Nevertheless, current studies more strongly support the idea that retrovirus-induced spongiform pathogenesis is mediated instead by unique Env protein structures unrelated to receptor binding and entry, especially since ecotropic Env pseudotyping would preclude post-entry amphotropic Env-receptor interactions.

The results presented here indicate that neurotoxic Envs act at very low levels, akin to very potent bacterial toxins. The current results and prior NPC transplantation studies (Li et al., 2016), indicate that Env/virus does not directly harm the cells in which it is expressed, even though it may impact cellular differentiation. Infected astrocytes were observed with protoplasmic and fibrous morphologies, and were observed making intimate neuron cell body contacts and establishing blood vessel endfeet. Whether these cells fully integrate and establish themselves as part of the tripartite synapse on dendrites and cell bodies remains to be determined. However, given the ability of astrocytes to modulate neuronal voltage-gated calcium channels (Mazzanti and Haydon, 2003), and regulate neuronal excitability (Fellin et al., 2006, 2007; Gourine et al., 2010; Halassa and Haydon, 2010), their infection could explain the elevated intracelluar calcium levels in rebound neurons associated with hyperexcitability and loss of rebound firing noted in FrCasE-induced spongiosis (Li et al., 2014; Sivaramakrishnan and Lynch, 2017). Further dissection of this process in the simple MLV-based system, using these new visualization tools will accelerate our understanding of how retroviruses cause neurodegeneration. The discovery here that subdetectable viral Env expression levels in astrocytes appears capable of negatively altering CNS motor system neurophysiology has significant implications for the various human neurodegenerative diseases that have been linked to endogenous and exogenous viruses.

## Data Availability Statement

The datasets generated for this study are available on request to the corresponding author.

## Ethics Statement

The animal study was reviewed and approved by the Northeast Ohio Medical University Institutional Animal Care and Use Committee.

## Author Contributions

SC designed and executed the experiments, analyzed and interpreted the data, and wrote a draft of the manuscript. JD designed and executed the experiments, and analyzed and interpreted the data. AD and CL designed and executed the experiments, and interpreted the data. WL conceived the study, designed and executed the experiments, analyzed and interpreted the data, and revised and edited the manuscript.

## Conflict of Interest

The authors declare that the research was conducted in the absence of any commercial or financial relationships that could be construed as a potential conflict of interest.
